# Does inflammatory bowel disease promote kidney diseases: a mendelian randomization study with populations of European ancestry

**DOI:** 10.1186/s12920-023-01644-2

**Published:** 2023-09-26

**Authors:** Xingji Lian, Yiqin Wang, Shuyi Wang, Xiaohui Peng, Yanhui Wang, Yuyu Huang, Wei Chen

**Affiliations:** 1 Department of Nephrology, The First Affiliated Hospital, NHC Key Laboratory of Nephrology (Sun Yat-sen University), Guangdong Provincial Key Laboratory of Nephrology, Sun Yat-sen University, Guangzhou, 510080 China; 2 Department of Geriatrics, Guangzhou First People’s Hospital, School of Medicine, National Key Clinic Specialty, South China University of Technology, Guangzhou, 510180 China; 3 Department of Rheumatology, The First Affiliated Hospital, Sun Yat-sen University, Guangzhou, 510080 China

**Keywords:** Chronic kidney disease, Immunoglobulin A nephropathy, Inflammatory bowel disease, Causality, Mendelian randomization

## Abstract

**Background:**

This study aimed to investigate a causal relationship between IBD and multiple kidney diseases using two-sample Mendelian randomization (MR) analyses.

**Methods:**

We selected a group of single nucleotide polymorphisms (SNPs) specific to IBD as instrumental variables from a published genome-wide association study (GWAS) with 86,640 individuals of European ancestry. Summary statistics for multiple kidney diseases were obtained from the publicly available GWAS. Genetic data from one GWAS involving 210 extensive T-cell traits was used to estimate the mediating effect on specific kidney disease. Inverse-variance weighted method were used to evaluate the MR estimates for primary analysis.

**Results:**

Genetic predisposition to IBD was associated with higher risk of IgA nephropathy (IgAN) (OR, 1.78; 95% CI, 1.45–2.19), but not membranous nephropathy, diabetic nephropathy, glomerulonephritis, nephrotic syndrome, chronic kidney disease, and urolithiasis. CD4 expression on CD4 + T cell had a significant genetic association with the risk of IgAN (OR, 2.72; 95% CI, 1.10–6.72). Additionally, consistent results were also observed when IBD was subclassified as ulcerative colitis (OR, 1.38; 95% CI, 1.10–1.71) and Crohn’s disease (OR, 1.37; 95% CI, 1.12–1.68). MR-PRESSO and the MR-Egger intercept did not identify pleiotropic SNPs.

**Conclusions:**

This study provides genetic evidence supporting a positive casual association between IBD, including its subclassification as ulcerative colitis and Crohn’s disease, and the risk of IgAN. However, no casual association was found between IBD and other types of kidney diseases. Further exploration of IBD interventions as potential preventive measures for IgAN is warranted.

**Supplementary Information:**

The online version contains supplementary material available at 10.1186/s12920-023-01644-2.

## Introduction

The inflammatory bowel diseases (IBD), including Crohn’s disease and ulcerative colitis, are also immune-mediated chronic or flares of inflammatory activity of the gastrointestinal tract [1]. Kidney-related extra-intestinal manifestations are common in IBD, occurring in 4 to 23% of IBD patients [2]. A cohort study involving renal biopsies of IBD patients with renal diseases showed that out of 896 IBD patients, 218 patients (24.3%) exhibited renal involvement, among which 25.7% were diagnosed with amyloidosis, 16.1% with immunoglobulin A nephropathy (IgAN), and 14.7% with crescentic glomerulonephritis [3]. Furthermore, a recent large-scale retrospective cohort study utilizing the United Kingdom electronic records database, identified 910 cases of chronic kidney disease (CKD) among 17,807 patients in the IBD cohort during follow-up, and found that IBD was associated with increased risk of CKD [4]. However, whether kidney diseases present as potential complication remains uncertain. Patients with IBD are susceptible to drug-related nephrotoxicity due to the nephrotoxic nature of certain medications used in their treatment, such as nonsteroidal anti-inflammatory drugs, methotrexate, and 5-aminosalicylic acid, and others [5, 6]. Distinguishing renal damage caused by nephrotoxic drugs or that resulting from the pathogenesis of IBD is challenging. Moreover, previous studies evaluating the association between IBD and kidney diseases had yielded conflicting results, likely due to the potential influence of residual confounding and reverse causation of observational studies [4, 7, 8]. Therefore, it’s essential to investigate whether there is a causal relationship between IBD and risk of renal diseases.

Mendelian randomization (MR) is an analytical approach used to infer causal associations between a modifiable exposure or risk factor and a clinically relevant outcome by integrating summary statistics from genome-wide association study (GWAS) [9, 10]. Based on the Mendel’s second law, genetic variants are randomly allocated in the population during gamete formation. The instrumental variables involved in the exposure will affect the outcomes proportionally if the exposure is causal. MR approach enables us to minimize the impact of confounding factors (e.g., environment) and reverse causation, serving as a mimic of the randomization in randomized controlled trial. This study conducted a two-sample MR analysis to examine the causal association between genetically predicted IBD and the risk of multiple kidney diseases. We aimed to evaluate which type of kidney disease primarily reflect this causal relationship.

## Methods

### Data sources

We used summary data on GWAS obtained from the International Inflammatory Bowel Disease Genetics Consortium (IIBDGC) [11]. It was a multi-ethnic large-scale genome-wide or Immunochip genotype data, in which we only included the extended cohort of 86,640 European individuals to identify new IBD risk loci and compared the genetic architecture of IBD susceptibility [11]. Beyond that, we also investigated the same consortium of Crohn’s disease and ulcerative colitis as subtypes of IBD with 20,550 and 17,647 cases vs. 41,642 and 47,179 controls, respectively. Regarding the multiple kidney diseases, including IgAN, membranous nephropathy, glomerulonephritis, diabetic nephropathy, nephrotic syndrome, chronic kidney disease, and kidney/ ureter/ bladder stone, the summary statistic were obtained from publicly available summary-level data of previously published GWAS and restricted to European descent [12–14]. We also retrieved the summary data from MR Base database from Medical Research Council Integrative Epidemiology Unit (MRC-IEU) and these datasets have already undergone the recommended quality control processes as previously described [16]. The extensive T-cell traits were derived from the SardiNIA project composed of GWAS data, which included 210 subtypes in the T-cell panel and cell marker expression levels on different T cells [15] The summary statistics could be archived in the IEU GWAS datasets (https://gwas.mrcieu.ac.uk). Table 1 shows the characteristics of all GWAS data for the exposure and outcome.

**Table 1:** Information on GWASs for exposure and outcome

Traits	Cases	Controls	Year	Number of SNPs	Sample size	Population	PMID/Consortium
**IgA nephropathy**	977	4,980	2010	278,077	5,957	European	20,595,679
**Membranous nephropathy**	2,150	5,829	2020	5,327,688	7,979	European	32,231,244
**Glomerulonephritis**	4,613	214,179	2021	16,380,466	218,792	European	IEU GWAS datasets
**Nephrotic syndrome**	480	214,619	2021	16,380,437	215,099	European	IEU GWAS datasets
**Diabetic nephropathy**	3,283	181,704	2021	16,380,336	184,987	European	IEU GWAS datasets
**Chronic kidney disease**	12,385	104,780	2016	2,191,877	117,165	European	26,831,199/ CKDGen
**Urolithiasis**	3,625	459,308	2018	9,851,867	462,933	European	MRC-IEU
**210 kinds of T-cell traits and markers**	-	-	2018	10,534,735	3,757	European	3,292,928
**Inflammatory bowel disease**	38,155	33,977	2015	157,116	86,640	European	26,192,919/ IIBDGC
**Crohn’s disease**	20,550	41,642	2015	124,888	62,192	European	26,192,919/ IIBDGC
**Ulcerative colitis**	17,647	47,179	2015	156,116	64,826	European	26,192,919/ IIBDGC

### Assumptions for instrumental variable selection

The genetic instrumental variable selection used in MR analysis was performed based on following three fundamental assumptions [17, 18]. Firstly, selected instrumental variables should be powerfully associated with exposure. The F statistic was used to evaluate the strength of the relationship between instrumental variables and exposure [16]. The formula of F statistic is expressed as F = R^2^ (n-k-1)/[k× (1-R^2^)]. R^2^ refers to the cumulative explained variance of selected single-nucleotide polymorphisms (SNPs) on IBD and is defined as R^2^ = 2 × effect allele frequency (EAF) × (1-EAF) × b^2^. EAF is the effect allele frequency and b is the estimated genetic effect; n is the sample size; and k represent the number of selected instrumental variables. If the resulting F value > 10, it indicates a strong correlation between instrumental variables and exposure, thus minimizing bias caused by weak instrumental variables. Secondly, instrumental variables should be independent of confounders that influence both the exposure and outcome. Third, selected instrumental variables should affect outcomes only through exposure, and not via alternative pathways. This implies the absence of a horizontal pleiotropy effect between instrumental variables and outcome. MR-Egger regression was performed to identify potential horizontal pleiotropy pathways [17].

**Fig. 1:**
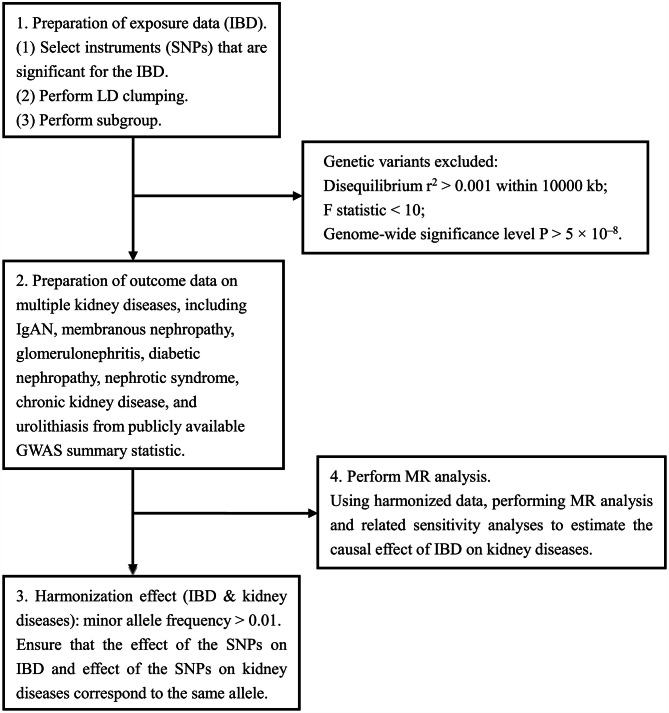
Flowchart describing the whole procedure. IBD: Inflammatory bowel disease; SNPs: single-nucleotide polymorphisms; LD: minor allele frequency; MAF: minor allele frequency; IgAN: IgA nephropathy; GWAS: genome-wide association study; MR: Mendelian randomization

In the present study, SNPs significantly associated with IBD were selected as genetic instrumental variable, which met the F-statistic > 10. To avoid the potential bias caused by strong linkage disequilibrium (LD) among the selected instrumental variables, we performed LD clumping process using an r^2^ cutoff of 0.001 and a clumping distance = 10,000 kb. We systematically gathered information on effect allele (EA), EA frequency, effect sizes (**β**), _S. E_. and *P*-value for further analysis. To ensure data consistency, we harmonized the respective exposure and outcome datasets using effect allele frequencies. Additionally, we removing palindromic SNPs with minor allele frequency (MAF) > 0.01. Figure 1 displays a flowchart illustrating the whole procedure.

### Statistical analysis

We conducted a two-sample MR in accordance with the Strengthening the Reporting of Observational Studies in Epidemiology-Mendelian Randomization (STROBE-MR) guidelines [19]. The causal effects of IBD on kidney diseases were estimated using the inverse-variance weighted (IVW) method for primary analysis. Moreover, to assess the robustness of IVW results, we compared them with other MR methods including MR-Egger, weighted median, simple mode, and weighted mode estimation. A consistent direction of estimates across all the MR methods increased confidence of causal relationship. The IVW method is essentially a meta-analysis method that requires all selected SNPs to be valid instrumental variables, with the intercept limited to zero. In the IVW method, a fixed-effect model was used when there was no heterogeneity (Cochrane’s Q *P*-value > 0.05), and a random-effect model was used otherwise. MR-Egger could be significantly influenced by outlying genetic variables, resulting in imprecise estimates, but it can still provide unbiased estimates even if there exist all invalid selected instrumental variables. The weighted median approach takes the median effect of all selected instrumental variables to provide accurate estimates with the prerequisite that at least half of SNPs are valid instrumental variables. The weighted mode approach remains still valid even if the other instrumental variables do not meet the requirements of MR method for causal inference. The MR-Egger regression was conducted to detect and adjusts for potential horizontal pleiotropic effects. Moreover, Cochran’s Q statistic was used to assess heterogeneity among the estimates from each SNP. The leave-one-out sensitivity analysis was performed to verify individual SNP that influenced the association disproportionately and to evaluate the stability of effect sizes. We also performed the Mendelian randomization pleiotropy residual sum and outlier (MR-PRESSO) test to estimate and correct outliers of instrumental variables with horizontal pleiotropic effects. Post-hoc power calculation for the main analyses was performed using mRnd website (http://cnsgenomics.com/shiny/mRnd/) [20]. Statistical analyses were conducted in R software (version 4.0.2; using the “TwoSampleMR”, “MendelianRandomization” and “MR-PRESSO” R package; R Foundation for Statistical Computing, Vienna, Austria). Two-tailed *P* < 0.05 was considered statistically significant.

## Results

### Genetic variants selection

The current study evaluated the causal effect of genetically predicted IBD on multiple kidney diseases. After removing SNPs with LD (r^2^ > 0.001 within 10,000 kb), palindromic SNPs (*P* > 5 × 10^8^ and F < 10), and duplicated SNPs, we finally selected 67 to 129 instrumental SNPs for each trait. Among all selected SNPs, 67 independent and significant SNPs were associated with IgAN (cases = 977), 108 associated with membranous nephropathy (cases = 2,150), 129 associated with glomerulonephritis (cases = 4,613), 79 associated with nephrotic syndrome (cases = 480), 129 associated with diabetic nephropathy (cases = 3,283), 82 associated with CKD (cases = 12,385), and 120 associated with urolithiasis (cases = 3,625). Detailed information on the genetic variants, including SNPs, EA, EA frequency, effect sizes on IBD, ulcerative colitis, and Crohn’s disease and multiple kidney diseases is provided in **Additional file 1: Table **S1–S9.

### Estimates of causal effect of IBD on kidney diseases

**Fig. 2:**
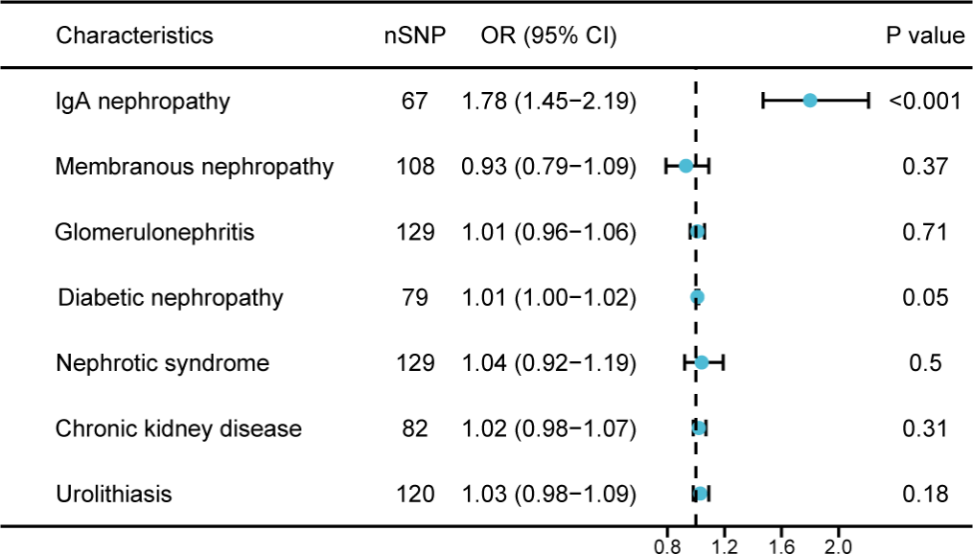
Forrest plots for associations of genetically predicted IBD with risk of kidney diseases SNP: single nucleotide polymorphism; OR: odds ratio; CI: confidence interval

The results of IVW with a fixed-effect model estimates showed a positive correlation between the genetically predicted IBD and the risk of IgAN [odds ratio (OR) 1.78 (95% confidence interval (CI) 1.45–2.19), *P* < 0.001]. However, no significant genetic association was detected between IBD and risk of membranous nephropathy (OR, 0.93; 95%CI, 0.79–1.09), glomerulonephritis (OR, 1.01; 95%CI, 0.96–1.06), diabetic nephropathy (OR, 1.01; 95%CI, 1.00–1.02), nephrotic syndrome (OR, 1.04; 95%CI, 0.92–1.19), chronic kidney disease (OR, 1.02; 95%CI, 0.98–1.07), and urolithiasis (OR, 1.03; 95%CI, 0.98–1.09) **(**Fig. 2**)**.

### Estimates of causal effect of IBD and subclassification on IgAN

**Fig. 3:**
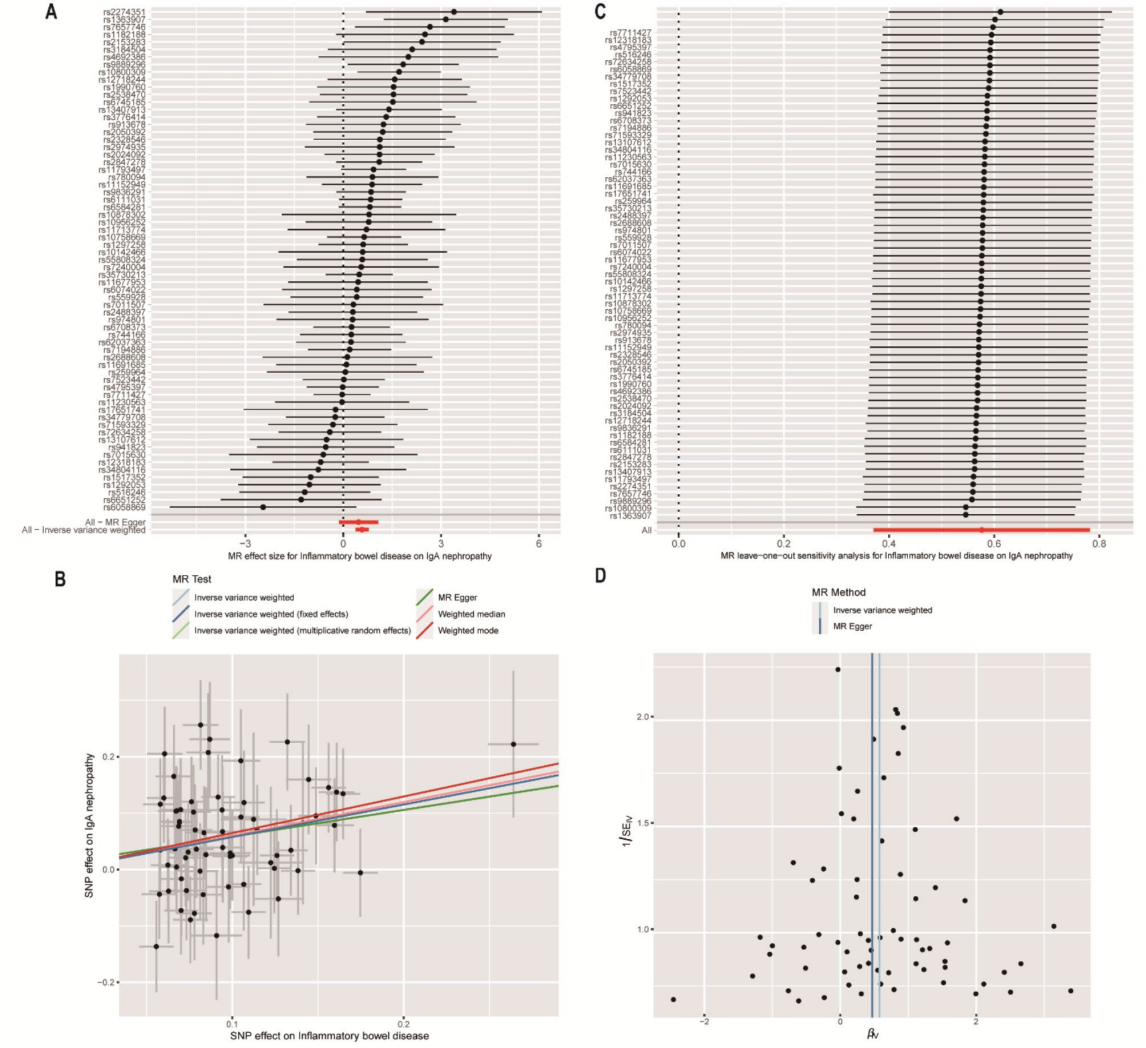
(**A**) Forest plot; (**B**) Sensitivity analysis; (**C**) Scatter plot; (**D**) Funnel plot of the effect of IBD on IgAN

We further compared with other MR methods to evaluate the robustness of IVW results of causal effect of IBD and subclassification on IgAN. The MR estimates of weighted median [OR 1.82 (95% CI 1.33–2.49), *P* < 0.001] and weighted mode methods [OR 1.91 (95% CI 1.16–3.14), *P* = 0.01] also showed consistent results **(**Table 2; Fig. 3A and B**)**. Post hoc power calculations indicated that the study had sufficient statistical powered (.100%) to detect an assuming of the real causal OR of 1.78 for the risk of IgAN with a total sample size of 5957 (19.6% ratio of IgAN case to control) and the significance level α of 0.05.

**Table 2:** Causal associations between genetically determined IBD level and IgAN

Exposure- outcome		Causal estimate			
	Method	SNP	OR	95% CI	*P* value
**IBD**	Inverse variance weighted*	67	1.78	1.45–2.19	< 0.001
	Weighted median	67	1.82	1.33–2.49	< 0.001
	Weighted mode	67	1.91	1.16–3.14	0.01
	Simple mode	67	1.66	0.86–3.20	0.14
	MR Egger	67	1.60	0.88–2.93	0.13
	Test for Heterogeneity: *P* = 0.53 (MR-Egger) and *P* = 0.56 (IVW)				
	Test for Horizontal pleiotropy: MR-Egger intercept = 0.0112, se = 0.031, *P* = 0.72				
	MR-PRESSO global test: *P* = 0.67				

^*^Inverse variance weighted (fixed-effect) method. IBD, Inflammatory bowel disease; IgAN: IgA nephropathy; SNPs: single-nucleotide polymorphisms; MR: Mendelian randomization; IVW: inverse-variance weighted; MR-PRESSO, Mendelian randomization pleiotropy residual sum and outlier

The genetic changes in the subclassification of IBD as ulcerative colitis, were also positively correlated with the risk of IgAN, which were showed in IVW based on a fixed-effect model [OR 1.38 (95% CI 1.10–1.71), *P* = 0.005; **Additional file 1: Table S10, Additional file 1: Fig.**S1A–S1B]. The results of IVW with a random-effect model showed that the genetic changes in Crohn’s disease were positively correlated with the risk of IgAN [OR 1.37 (95% CI 1.12–1.68), *P* = 0.002; **Additional file 1: Table S11, Additional file 1: Fig.**S2A–S2B]. However, no significant causal association between ulcerative colitis or Crohn’s disease and IBD were found in MR-Egger, weighted median and weighted mode methods **(Additional file 1: Table S10–S11)**.

### Estimates of causal association mediated by T cells traits and IgAN

The detailed information regarding the MR result, including EA, EA frequency, effect sizes on specifical T-cell traits and IgAN is shown in **Additional file 1: Table S12**. After harmonizing the respective T-cell phenotype exposure and outcome datasets, we identified 42 out of a total of 210 T-cell phenotypes had valid instrumental variables. Subsequently analyzing the 42 T-cell phenotypes in MR analysis, we only observed a significant genetic association between CD4 expression on CD4 + T cells and IgAN [OR 2.72 (95% CI 1.10–6.72), P = 0.029]. No statistically significant differences were observed for other T-cell traits **(Additional file 1: Table S13)**.

### Sensitivity analyses

Sensitivity analyses using the leave-one-out associations approach suggested that the causal association was not biased by potential driving SNP (Fig. 3C, **Additional file 1: Fig.**S1C, and Additional file 1: Fig. S2C). The horizontal pleiotropy between instrumental variables and outcome was assessed by MR-Egger regression, and the results indicated the predicted effect sizes of IBD, ulcerative colitis, and Crohn’s disease on IgAN were comparable and consistent in the direction [IBD: intercept = 0.0112 (_SE_ = 0.031), *P* = 0.75, Fig. 3B], [ulcerative colitis: intercept = -0.007 (_SE_ = 0.04), *P* = 0.87, **Additional file 1: Fig.**S1B], and [Crohn’s disease: intercept = -0.009 (_SE_ = 0.038), *P* = 0.82, **Additional file 1: Fig.**S2B]. Besides, the MR-PRESSO method did not detect outlying SNPs causing horizontal pleiotropy [*P*-value for MR-PRESSO global test = 0.67 (IBD), 0.99 (ulcerative colitis), and 0.86 (Crohn’s disease)]. Furthermore, no heterogeneity of IBD and ulcerative colitis on IgAN was found in IVW and MR Egger method using the Cochran’s Q test [Cochran’s Q = 62.958, *P* = 0.53 (MR-Egger) and *P* = 0.56 (IVW), Fig. 3D] and [Cochran’s Q = 45.04, *P* = 0.10 (MR-Egger) and *P* = 0.17 (IVW), Additional file 1: **Fig.**S1D]. However, there was significant heterogeneity in Crohn’s disease [Cochran’s Q = 93.60, *P* = 0.01 (MR-Egger) and *P* = 0.02 (IVW), **Additional file 1: Fig.**S2D].

## Discussion

The current study revealed a positive correlation between IBD and the risk of IgAN, and CD4 expression on CD4 + T cell could involve the causal effects. Additionally, the positive associations were also observed when subclassifying IBD into ulcerative colitis and Crohn’s disease. However, no statistical differences were found between IBD and risk of membranous nephropathy, glomerulonephritis, diabetic nephropathy, nephrotic syndrome, chronic kidney disease, and urolithiasis.

A observational study evaluated 83 kidney biopsy specimens from patients with IBD and found that IgAN was the most prevalent diagnosis (24%). This prevalence was significantly higher compared to all native non-IBD kidney biopsy specimens (24% versus 8%) [21]. Another retrospective analysis of renal biopsies done for IBD patients in Egypt revealed that out of 896 IBD patients, 218 (24.3%) developed renal complications, with IgAN being the second most common renal pathological diagnosis (16.1%) [3]. Additionally, a recent population-based cohort study including 3963 biopsy-verified IgAN patients and 19,978 matched controls, confirmed a correlation between IgAN and IBD, and further that a diagnosis of IBD among patients with IgAN were more likely than to progress into end-stage kidney disease than those without IBD [22]. To our knowledge, apart from the aforementioned cohort studies [3, 21, 22], the association between IgAN and IBD has mainly been described through single earlier case reports [23–26]. In present MR analysis, the use of SNPs as proxies of IBD allowed us to minimize the impact of confounding factors and inverse causality, which are common in observational study [27]. Our study provides genetic-level support for the positive association between IBD and the risk of IgAN, but not other types of kidney diseases. This finding aligns with the recent announcement by the Unite State Food and drug administration (FDA) and clinical trials that budesonide in oral capsules, designed to release active compounds upon reaching the distal ileum, is primarily intended for the treatment of adult primary IgA nephropathy, rather than other types of kidney diseases [28–30].The underlying mechanisms involved in deposition of IgA in glomerular of patients with inflammation of the intestinal mucosa remain to be unraveled. Recent advances have highlighted the important role of the gut-kidney axis in pathophysiologic connection between IBD and IgAN [31–33]. Firstly, it has been observed that most IBD patients experience hyperactivity of IgA1-secreting cells in the lesions of intestinal mucosa, which leads to an imbalance in the of immunoglobulin production [4, 34]. This imbalance could be due to the impair of mucosal integrity, which acts as a barrier to antigenic stimulation. Additionally, an increase in intestinal permeability and an inadequate response to the microbiota (rather than specific taxa), potentially driven by host cytokines like B-cell activating factor, such as B-cell activating factor [33]. Furthermore, recent GWAS have identified 9 genetic variants that control composition of gut microbiota may be associated with susceptibility to IgAN. This finding suggests that host genetics can influence gut microbiota in IgAN [35]. Interestingly, our results indicated a positive correlation between CD4 expression on CD4 + T cell and the risk of IgAN, among 210 extensive T-cell traits. Previous study have shown that serum levels of IgA strongly correlate with the percentage of CD4 + CD45RO + cells in peripheral blood in patients with IgAN, indicating that CD4 + T cells might also play a role in abnormal IgA1 glycosylation process [36–38]. Therefore, it might be worth exploring clinical trials with drugs that target the imbalance in the CD4 + T cell compartment.

### Limitations

This study has several limitations. First, we cannot determine the extent of overlapping participants involved between data sources for SNPs-exposure and outcome consortia in two sample MR analyses. However, to minimize the potential deviation from participant overlap, we employed F statistic and instrumental variables filtering procedures. Second, all analyses were based from European ancestry individuals, and it is necessary to conduct further GWAS studies in other ethnicities to validate the results. Third, we investigated the casual effect of only one T-cell traits on the risk of IgAN, and it is possible that there were insufficient numbers of SNPs available to determine the causal effect. Hence, caution is needed when interpreting the results. Finally, we were unable to completely exclude the possibility of other direct causal pathway from the IBD-predisposing genetic variants to IgAN. To note, the subgroup analyses demonstrated a significant heterogeneity within the population with Crohn’s disease. This suggests the presence of potential variations in the validity of SNPs and false positive results, which can be attributed to the constraint imposed by the limited sample size. Nevertheless, both the MR-Egger intercept and MR-PRESSO analyses showed little heterogeneity among instrumental variables of IBD and minimal directional horizontal pleiotropy, suggesting a lower likelihood of bias.

## Conclusions

The current MR study provides robust evidence supporting the causal association between genetic susceptibility to IBD and an increased risk of IgAN, as well as its specific subtypes including ulcerative colitis and Crohn’s disease. However, there is insufficient evidence to establish a causal link between IBD and other kidney diseases such as, membranous nephropathy, glomerulonephritis, diabetic nephropathy, nephrotic syndrome, chronic kidney disease, and urolithiasis. Further investigation using updated data from large-scale genetic studies is necessary to validate and reinforce the findings.

### Electronic supplementary material

Below is the link to the electronic supplementary material.

Supplementary Material 1

Supplementary Material 2

## Data Availability

Only publicly available data were used in this study, and data sources and handling of these data were described in the Materials and Methods and supplementary files. The datasets analyzed for this study are available on the website (https://gwas.mrcieu.ac.uk). Further inquiries can be directed to the corresponding author Wei Chen.

## Abbreviations

IgAN: Immunoglobulin A nephropathy
ESKD: End-stage kidney disease
IBD: Inflammatory bowel disease
MR: Mendelian randomization
GWAS: Genome-wide association study
SNPs: Single-nucleotide polymorphisms
EAF: Effect allele frequency
MAF: Minor allele frequency
LD: Minor allele frequency
EA: Effect allele
IVW: Inverse-variance weighted
MR-PRESSO: Mendelian randomization pleiotropy residual sum and outlier
OR: Odds ratio
CI: Confidence interval
