# Supplementary material for: Does inflammatory bowel disease promote kidney diseases: a mendelian randomization study with populations of European ancestry
Source: BMC Med Genomics. 2023 Sep 26;16:225. doi: 10.1186/s12920-023-01644-2 (PMC10521387; doi:10.1186/s12920-023-01644-2)
Supplement: Supplementary file 1 — Supplementary Material 1 [file 12920_2023_1644_MOESM1_ESM.docx]

**Investigating the Causal Associations between Inflammatory Bowel Disease and Risk of Kidney Diseases: A Two-Sample Mendelian Randomization Analysis**

**Table of contents**

|  |  | | **Page** | |
| --- | --- | --- | --- | --- |
| Supplemental Figure S1 | | 1. Forest plot of the effect of ulcerative colitison on IgAN 2. Scatter plot of the effect of ulcerative colitis on IgAN 3. Sensitivity analysis plot of the effect of ulcerative colitis on IgAN 4. Funnel plot of the effect of ulcerative colitis on IgAN | | 1 |
| Supplemental Figure S2 | | 1. Forest plot of the effect of Crohn's disease on IgAN 2. Scatter plot of the effect of Crohn's disease on IgAN 3. Sensitivity analysis plot of the effect of Crohn's disease on IgAN 4. Funnel plot of the effect of Crohn's disease on IgAN | | 2 |


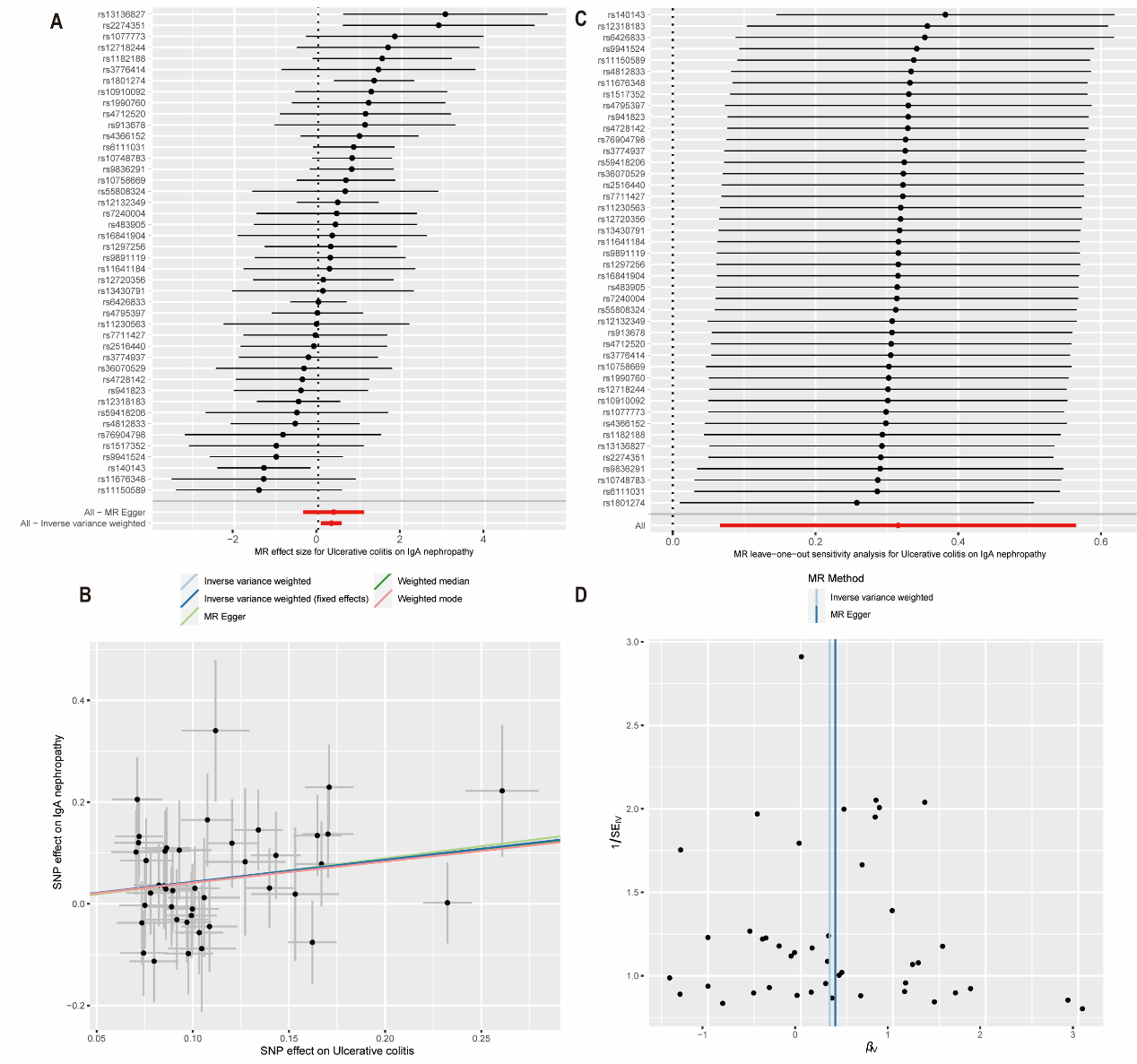


**Supplemental Figure S1.** Forest plot (A), sensitivity analysis (B), scatter plot (C) and funnel plot (D) of the effect of ulcerative colitis on IgAN


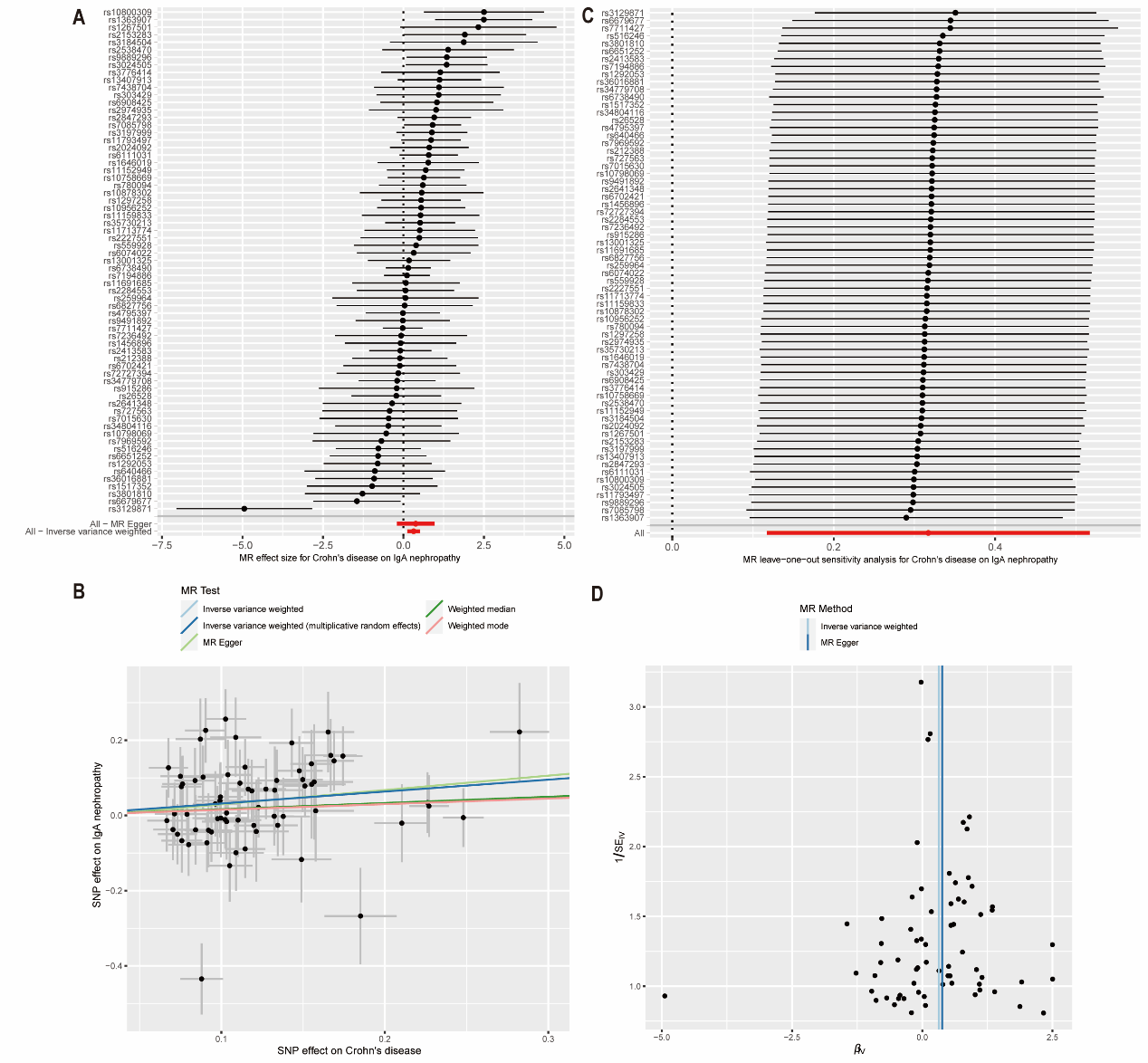


**Supplemental Figure S2.** Forest plot (A), sensitivity analysis (B), scatter plot (C) and funnel plot (D) of the effect of Crohn's disease on IgAN
